# Editorial: Neuroscience of posture and gait control: mechanisms, influencing factors and cognitive-motor retraining

**DOI:** 10.3389/fnhum.2023.1197022

**Published:** 2023-05-16

**Authors:** Tuhin Virmani, Olga M. Bazanova, Linda J. Larson-Prior

**Affiliations:** ^1^Department of Neurology, University of Arkansas for Medical Sciences, Little Rock, AR, United States; ^2^Department of Biomedical Informatics, University of Arkansas for Medical Sciences, Little Rock, AR, United States; ^3^State Research Institute of Neuroscience and Medicine, Novosibirsk, Russia; ^4^Department of Neurobiology and Developmental Sciences, University of Arkansas for Medical Sciences, Little Rock, AR, United States

**Keywords:** gait, balance, postural control, somatosensory integration, aging, neurologic disease

Postural and balance control systems integrate to maintain upright stance, while gait control pathways additionally integrate during walking. Visual, somatosensory and vestibular information from the environment feeds into these control pathways at the motor planning stages and provides continuous feedback to adapt movement to a changing environment (Figure 1). Learned motor pathways subserve complex motor behaviors, such as riding a bicycle, but cognitive control circuitry can override these learned pathways based on environmental inputs. During childhood, development of these processes occurs in response to external stimuli and trial and error performance of these tasks to fine tune the integration of these pathways.

**Figure 1:**
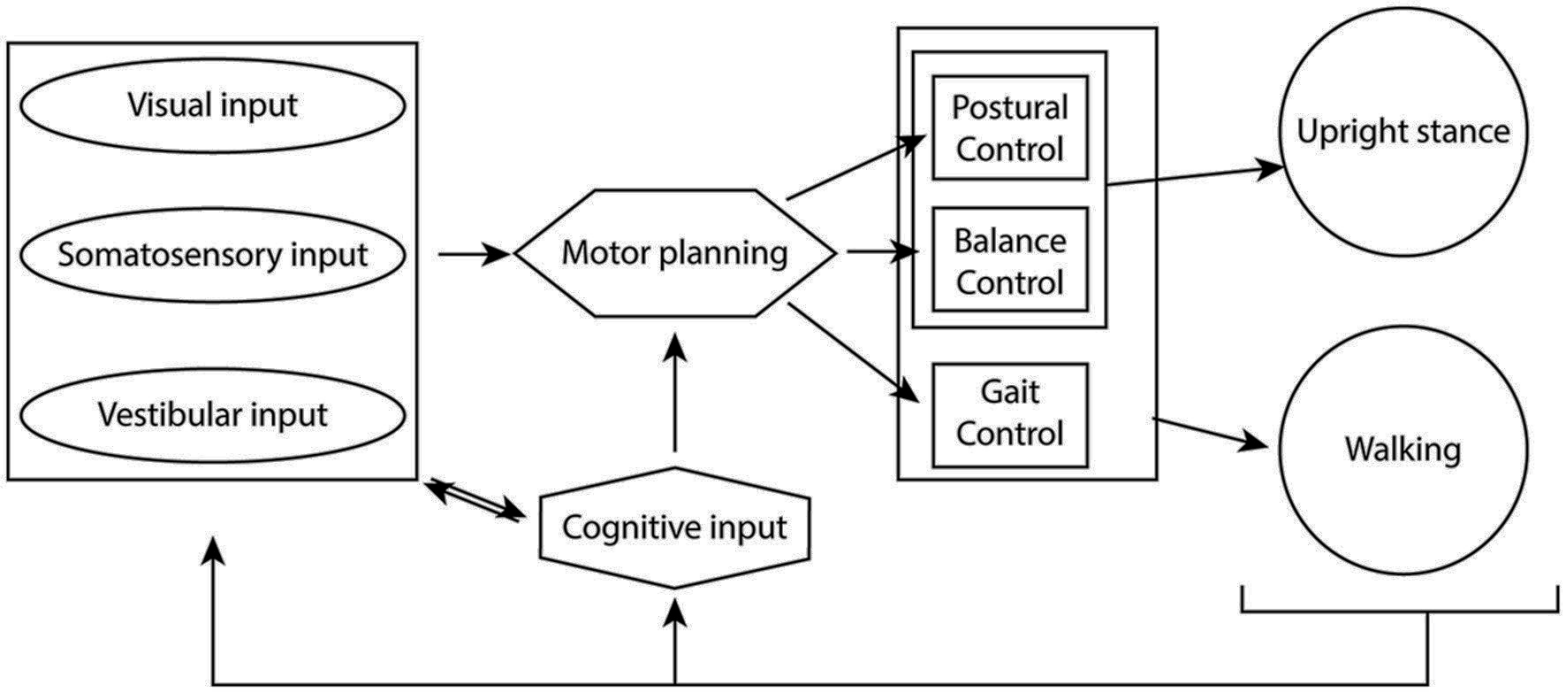
Cognitive-sensory-motor integration to maintain upright stance and coordinated walking. Visual, somatosensory and vestibular inputs from the environment integrate with cognitive oversight to help plan motor output. Postural and balance circuitry maintain upright stance, while the act of walking also requires integration of gait control circuitry. The sensory inputs provide constant feedback to the system in order to maintain optimal gait, balance and postural control of movement.

These processes can be affected by disease at any developmental stage, impacting one or more control pathway in the central and peripheral nervous system. Sedentary behavior may result in loss of learned behaviors, thus requiring increased cognitive control of movement (Hausdorff et al., 2005). Aging may lead to slowed walking (Tian et al., 2017), requiring postural and balance mechanisms to adapt to the changing motor output and feedback sensory input (Paraskevoudi et al., 2018). Reduced gait speed is highly associated with increased fall risk (Verghese et al., 2009), dependency and mortality. Accumulating evidence also indicates that postural instability, reduced gait speed and fall risk are associated with poorer cognitive function (Camicioli et al., 1998; Kido et al., 2010; Pieruccini-Faria et al., 2020). Furthermore, it is becoming increasingly important to include the modulatory effects of affective, autonomic, metabolic and cardiovascular dysfunction on gait and postural control pathways.

To understand the impact of disease states on posture, balance and gait control, a better understanding of the development of these process and how they each may change over an individual's lifetime is needed. This will allow development of more targeted rehabilitation and neuromodulatory treatments to correct deficits in disease states.

In typically developing children Mani et al. use spatial margin of stability as an index of dynamic postural stability to show that dynamic postural stability was not mature until approximately the age of 10 and was associated with development of contralateral coordination in the limbs and trunk/pelvis twist coordination, previously reported to mature around age 5. Later maturation of complex coordinated control of axial twist and inter-limb coordination relies on maturation of cortical control systems while earlier maturation would suggest early development of spinal central pattern generators. In both typically developing children and children with Cerebral Palsy, Kim et al. explore the impact of external environmental changes in a walking task on muscle synergies (the groups of coactive muscles and time varying activation patterns that lead to movement). Under varied walking conditions that included preferred speed, variable speed and constrained width they find that these conditions altered the structure of muscle synergies in cerebral palsy but not typically developing children. This suggests that individualized physical therapy to adapt the different muscle synergies in cerebral palsy children could be beneficial.

In another study exploring muscle synergies, Oshima et al. used a split-belt treadmill walking paradigm in 12 healthy young male adults. Split-belt treadmills are used to assess adaptability of gait response and also in rehabilitation of asymmetric gaits. The authors found that by changing speed of the split belt, the adaptation responses in the faster and slower legs, were at the level of muscle synergies with differing recruitment and timing of activation patterns of the coactive muscle groups. This adaptation to split-belt treadmill walking at the muscle synergy level could be useful in designing future rehabilitation strategies.

Two studies explored fundamental processes related to postural stability. Watanabe and Higuchi asked a more fundamental question regarding the maintenance of postural stability. Using a “go-before-you-know” task in which participants initiated stepping before they knew which of two targets was the true target for their stepping foot, they suggest that mediolateral postural stability was more important in motor planning and, along with action costs, for maintaining postural stability. In their study, Kawasaki et al. explored the sympathetic response (ectodermal skin response, EDR) to task-specific perturbation in stance as a representation of multiple cognitive responses, including that of fear of falling. They report an early peak in the EDR response that supports the view that perturbation-induced EDR can represent multiple mental responses as a function of task, while EDR amplitude is related to the fear of falling. Interestingly, visual occlusion did not affect the EDR induced by perturbation in this study. Thus, this study elucidates the complex interplay of sensorimotor and cognitive mechanisms in response to perturbation in stance.

In two studies, virtual reality systems were used to explore dynamic control of balance under challenging conditions. Bzdúšková et al. used a virtual reality environment of an open air elevator with an unstable platform to study the psychological, autonomic (heart rate, EDR) and postural reactions to height in individuals with self-reported fear of heights. Participants with fear of heights had greater sympathetic activation and body postural sway (center of pressure) than those without. These findings could be used to develop technology to help those with fear of heights overcome these issues. In a second virtual reality study, Symeonidou and Ferris found that intermittent visual occlusions, using electrically controlled liquid crystal glasses, improved outcomes for dynamic balance control by 78% on the day of training and 60% 2 weeks after training. This intervention could therefore be helpful in reducing falls, in individuals with neurological or mechanical balance deficits.

One intervention study was reported in this Research Topic. In this study, Lacour et al. compared early (first 2 weeks) or later (5th and 6th weeks) vestibular rehabilitation in 69 people after a vertigo attack. The group that responded best to vestibular rehabilitation was the group with early intervention and high vestibulo-ocular reflex gains, independent of visual input. This suggests that early rehabilitation is beneficial in the correctly chosen patient.

In this Research Topic, studies address the complex interplay between cognitive and motor control networks in posture and gait in health and disease. Together, these studies point to the importance of individual responses to environmental perturbations of balance and gait when considering rehabilitative and therapeutic strategies in individuals with motor challenges while emphasizing the important role of cognitive function in modulation of motor control systems.

## Author contributions

TV and LL-P contributed to editorial review of published articles, writing, and revision of editorial. OB contributed to review of published articles and review of editorial. All authors contributed to the article and approved the submitted version.
